# Supplementary material for: Bead-Ejection Scenario in Electrospray Ionization of Multidomain Nucleic Acids
Source: Anal Chem. 2026 Feb 24;98(9):6547–55. doi: 10.1021/acs.analchem.5c04790 (PMC12980496; doi:10.1021/acs.analchem.5c04790)
Supplement: Supplementary file 1 [file ac5c04790_si_001.pdf]

Bead-Ejection Scenario in Electrospray Ionization of Multi-Domain Nucleic Acids

Debasmita Ghosh,[1] Frédéric Rosu[2,3] and Valérie Gabelica[1,3]*

[1] Univ. Bordeaux, INSERM, CNRS, ARNA, UMR 5320, U1212, F-33000 Bordeaux, France

[2] Univ. Bordeaux, CNRS, INSERM, IECB, UAR3033, US01, F-33600 Pessac, France

[3] School of Pharmaceutical Sciences, University of Geneva, Geneva, Switzerland (valerie.gabelica@unige.ch)

Supporting Information Table of Contents

Number	Description	Page
**Figure S1**	Melting curve of different 60-mers	3
**Figure S2**	ESI-MS spectra of G4Tn and TnG4Tn in high ionic strength (unfiltered data)	4
**Figure S3**	MS spectra of 7-, 8- ions of all oligonucleotides showing adducts	5
**Figure S4**	MS spectra of all high charge states of G4Tn showing adducts	6
**Figure S5**	MS spectra of all high charge states of TnG4 showing adducts	7
**Figure S6**	MS spectra of all high charge states of TnG4Tn showing adducts	8
**Figure S7**	MS spectra of all high charge states of NG showing adducts	9
**Figure S8**	MS spectra of 8- ion of G4Tn at F400 and F450	10
**Figure S9**	Arrival time distribution of G4Tn at 8- charge state	11
**Figure S10**	ESI-MS spectra of 60-mers at 50 mM ionic strength	12
**Figure S11**	ESI-MS spectra of 60-mers at 10- and 1-mM ionic strength	13
**Figure S12**	CCSD of TnG4Tn at various ionic strengths	14

**Figure S13**	Modelled structure of TnG4Tn	15
**Figure S14**	CCSD of NG at various ionic strengths	16
**Figure S15**	ESI-MS spectra of G4TnG4 at 150 and 50 mM ionic strength (unfiltered data).	17
**Figure S16**	MS spectra of all charge states of G4TnG4 showing adducts	18
**Figure S17**	Native supercharging of G4TnG4	19
**Figure S18**	Native supercharging of G4Tn	20
**Figure S19**	Native supercharging of TnG4Tn and NG	21
**Figure S20**	CIU experiments for G4Tn and TnG4Tn	22
**Figure S21**	CIU experiments for G4Tn	23
**Figure S22**	CIU experiments for TnG4	24
**Figure S23**	CIU experiments for TnG4Tn	25
**Figure S24**	CIU experiments for NG	26
**Figure S25**	CIU experiments for G4TnG4	27
**Figure S26**	CCS of detected peaks as a function of the charge states, recorded in native supercharging conditions	28

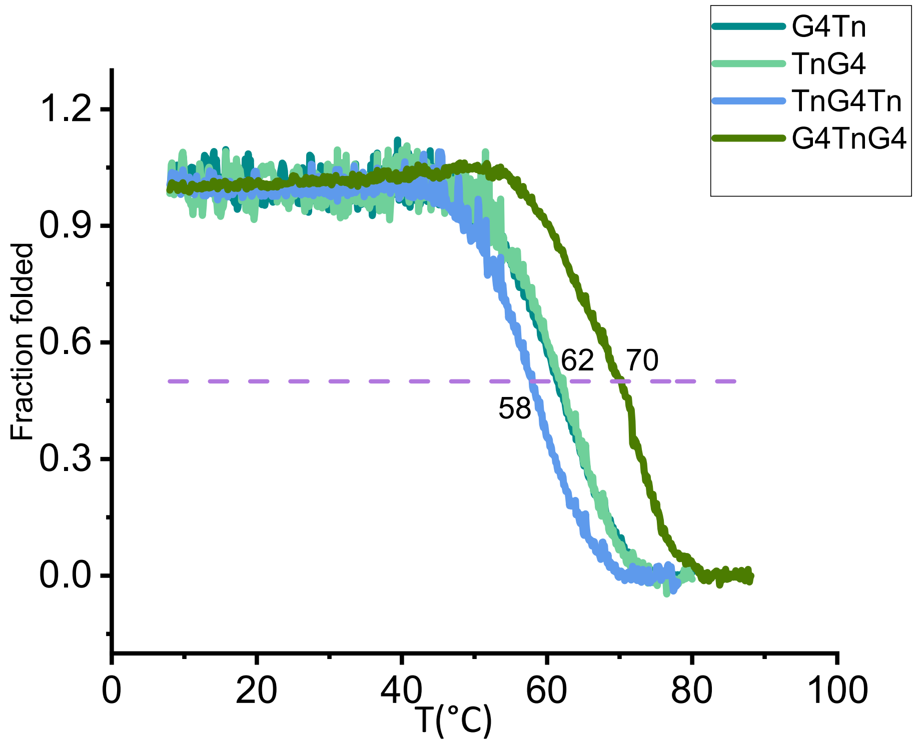

Figure S1: Melting curve of different 60-mer oligonucleotides at physiological ionic strength (150 mM aqueous NH_4OAc solution) showing that the structures are folded at room temperature.

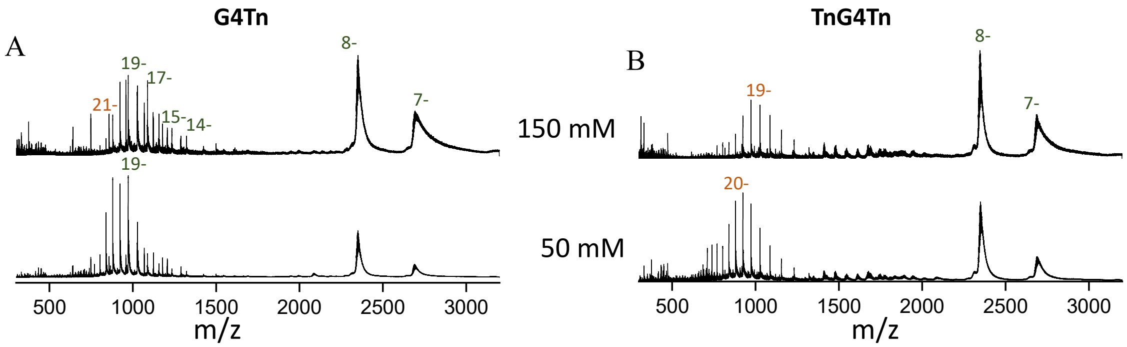

Figure S2: ESI-MS spectra of 60-mer oligonucleotides, G4Tn (A) and TnG4Tn (B) in 150 and 50 mM aqueous NH_4OAc (unfiltered data).

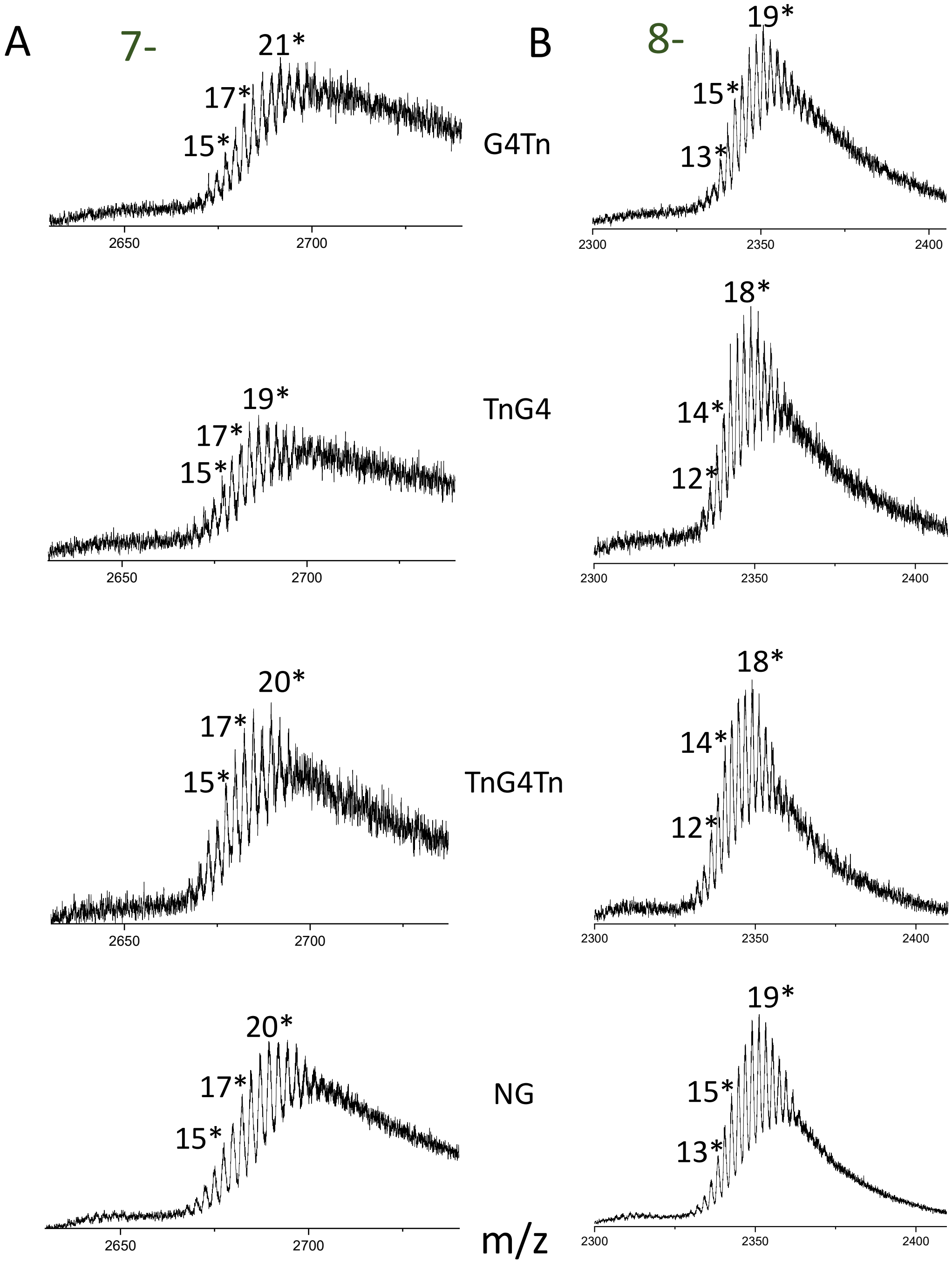

Figure S3: MS spectra of G4Tn, TnG4, TnG4Tn, and NG recorded at 320V exhibit multiple NH_4^+-adducts in the 7^- (A) and 8^- (B) charge states in 150 mM NH_4OAc. The numbers indicate the count of non-specific NH_4^+-adducts (marked with an asterisk *).

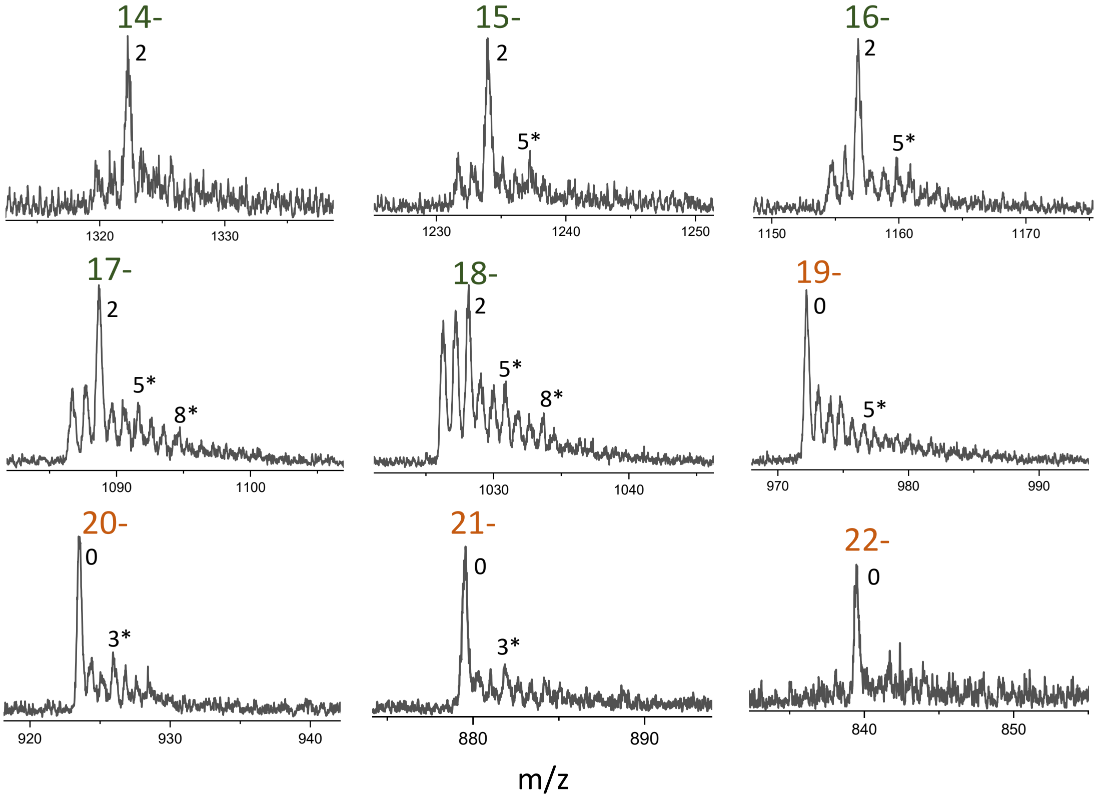

Figure S4: MS of G4Tn recorded at 320V show few NH_4^+-adducts at high charge states in 150 mM NH_4OAc. The numbers indicate the count of non-specific NH_4^+-adducts (marked with an asterisk *), and specific adducts (without asterisk).

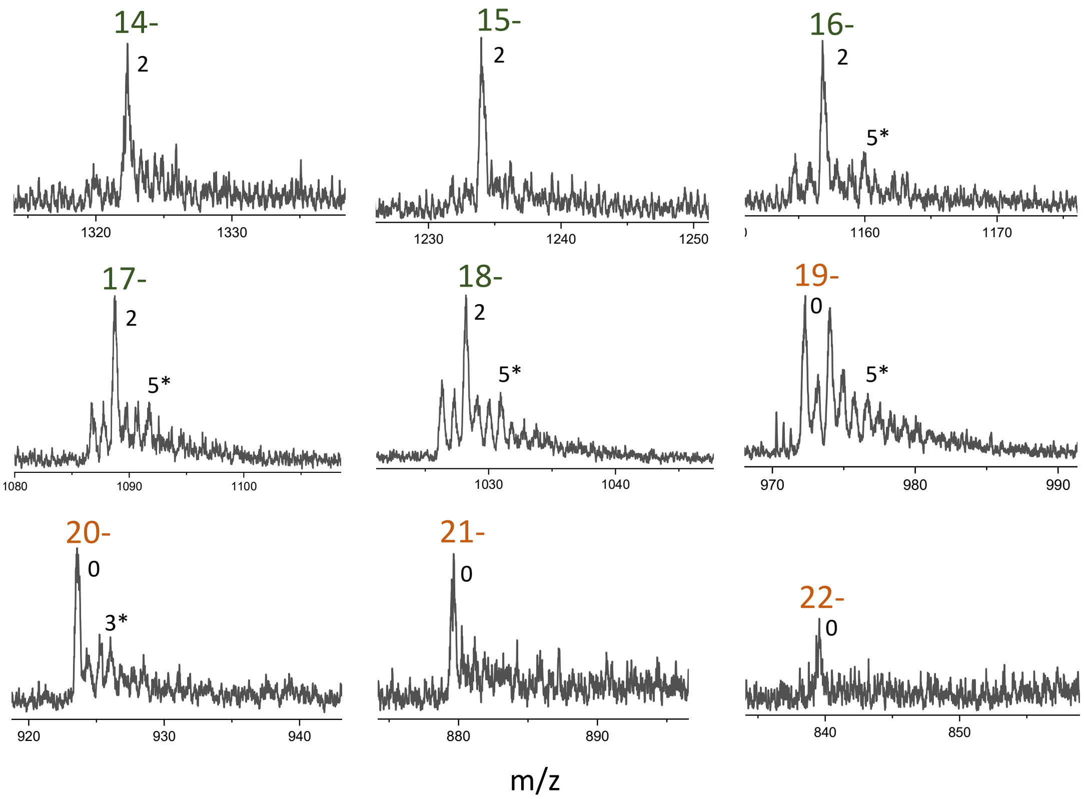

Figure S5: MS of TnG4 recorded at 320V show few NH_4^+-adducts at high charge states in 150 mM NH_4OAc. The numbers indicate the count of non-specific NH_4^+-adducts (marked with an asterisk *), and specific adducts (without asterisk).

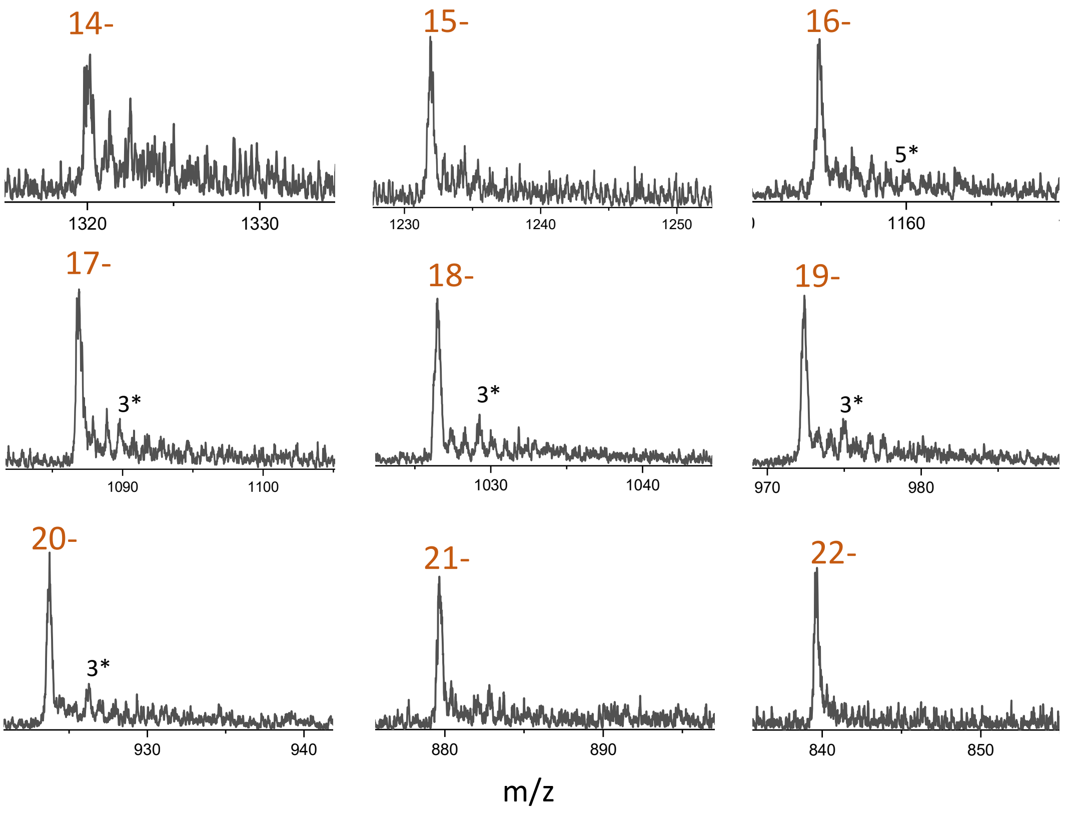

Figure S6: MS of TnG4Tn recorded at 320V show few NH_4^+-adducts at high charge states in 150 mM NH_4OAc. The numbers indicate the count of non-specific NH_4^+-adducts (marked with an asterisk *).

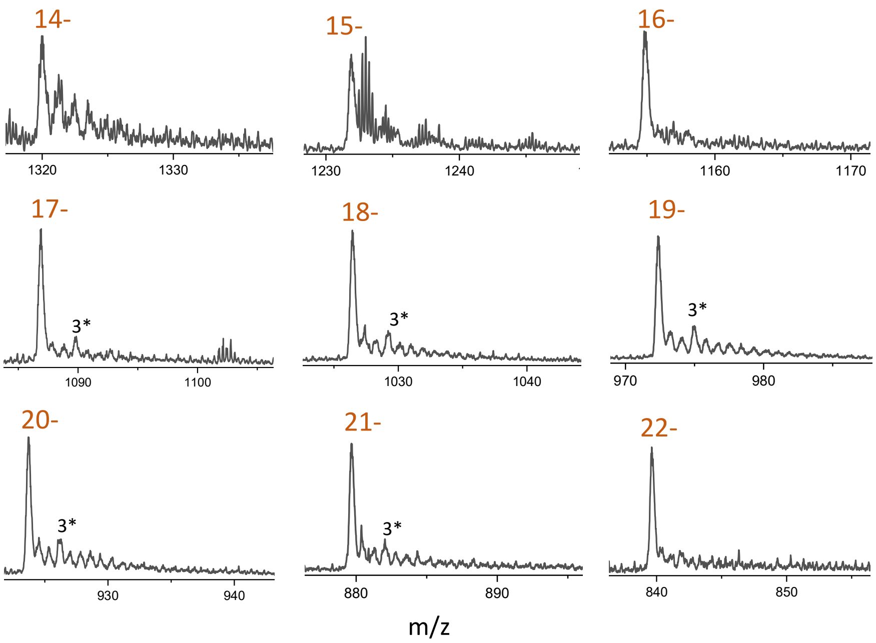

Figure S7: MS of NG recorded at 320V show few NH_4^+-adducts at high charge states in 150 mM NH_4OAc. The numbers indicate the count of non-specific NH_4^+-adducts (marked with an asterisk *).

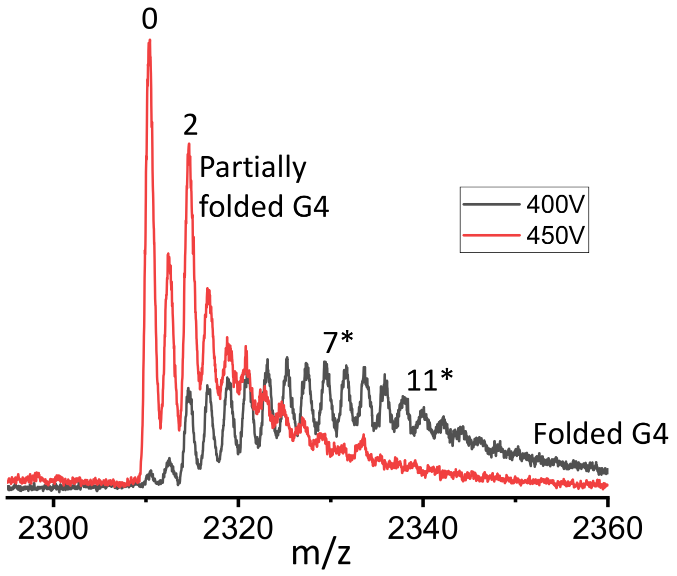

Figure S8: MS of G4Tn in 150 mM aqueous NH_4OAc show multiple NH_4^+-adducts at a low fragmentor voltage (400V) in the low charge state, 8-. The numbers indicate the count of specifically bound NH_4^+ ions, while number marked with an asterisk (*) denote non-specific NH_4^+-adducts. Increasing the fragmentor voltage to 450V improves the removal of nonspecific adducts but also causes the loss of intact G4 structures, even at the low-charge state (8−).

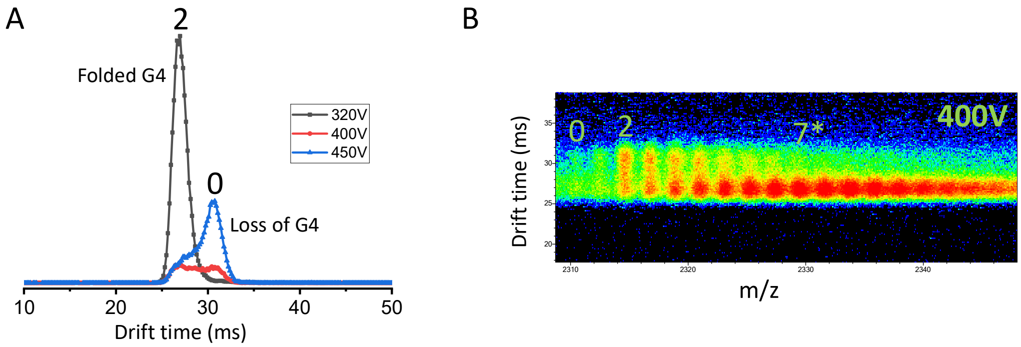

Figure S9: A) The arrival time distribution plot for G4Tn at an 8- charge state in 150 mM aqueous NH_4OAc illustrates the effect of varying the fragmentor voltages. At 320V (low voltage), the structure is fully folded, appearing as a single distribution. At 400V, two distributions emerge: one with two specifically bound NH_4^+ ions and another lacking NH_4^+ ions. At 450V (high voltage), the distribution without bound NH_4^+ ions dominates, indicating the loss of G-quartets. B) The 2D IMS plot shows multiple NH_4-adducts at a fragmentor voltage (400V) in the 8- charge state. The numbers indicate the count of specifically bound NH_4^+ ions, while number marked with an asterisk (*) denote non-specific NH_4^+-adducts.

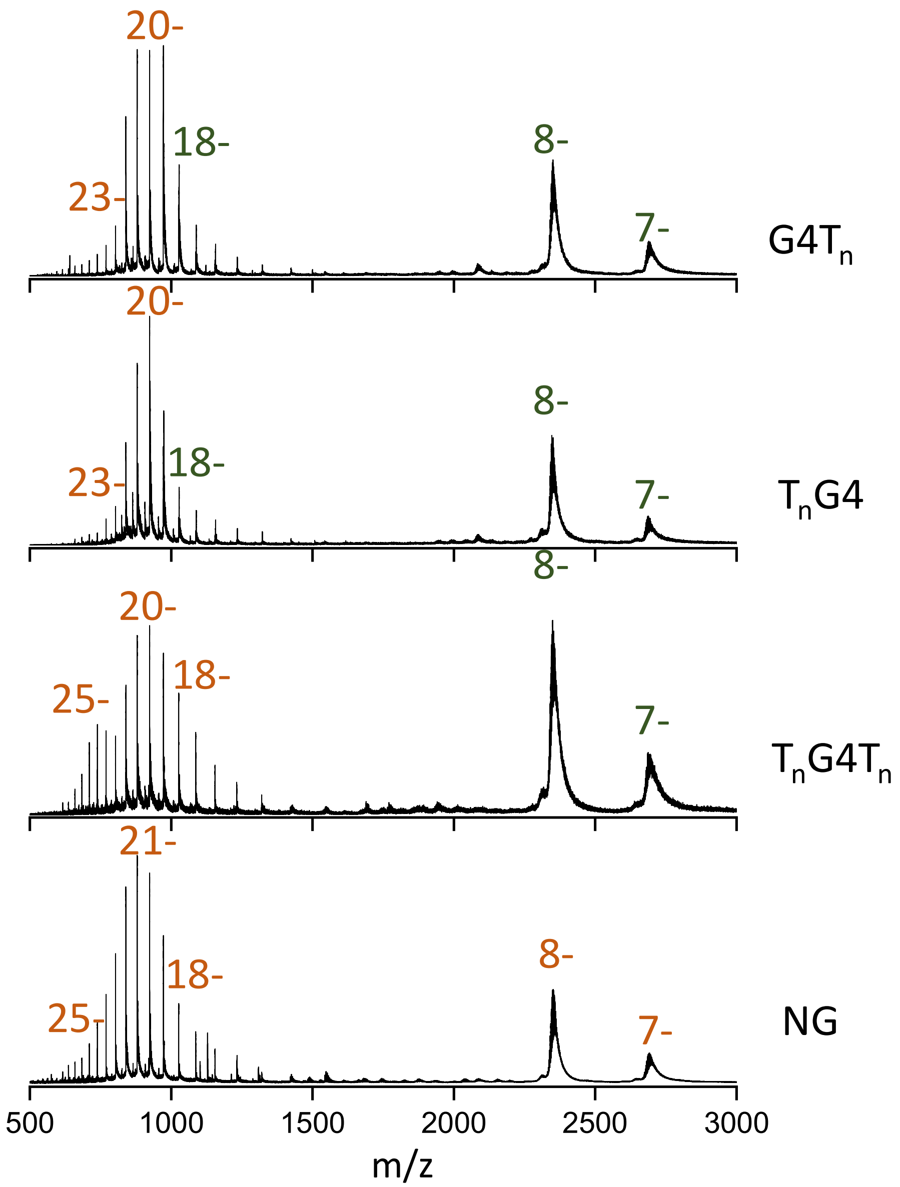

Figure S10: ESI-MS spectra of different 60-mer oligonucleotides (15 µM each) in 50 mM aqueous NH_4OAc.

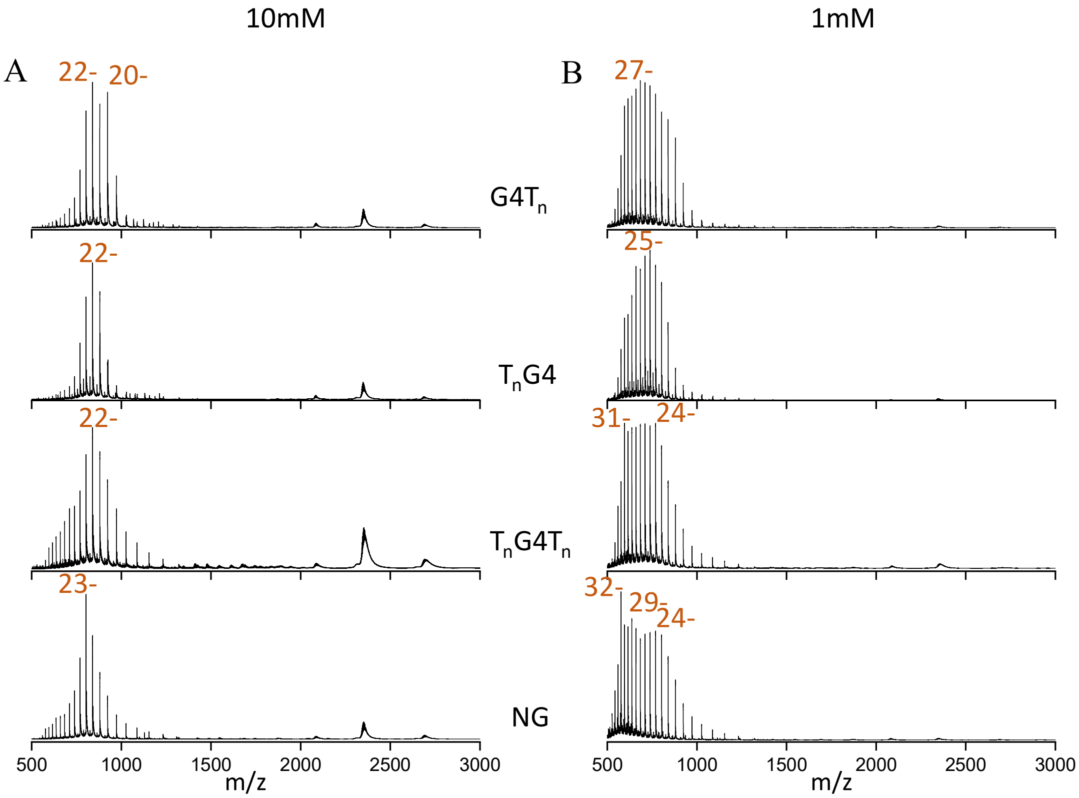

Figure S11: ESI-MS spectra of different 60-mer oligonucleotide structures (15 µM each) at low ionic strength, i.e., 10 (A), and 1 mM (B) aqueous NH_4OAc.

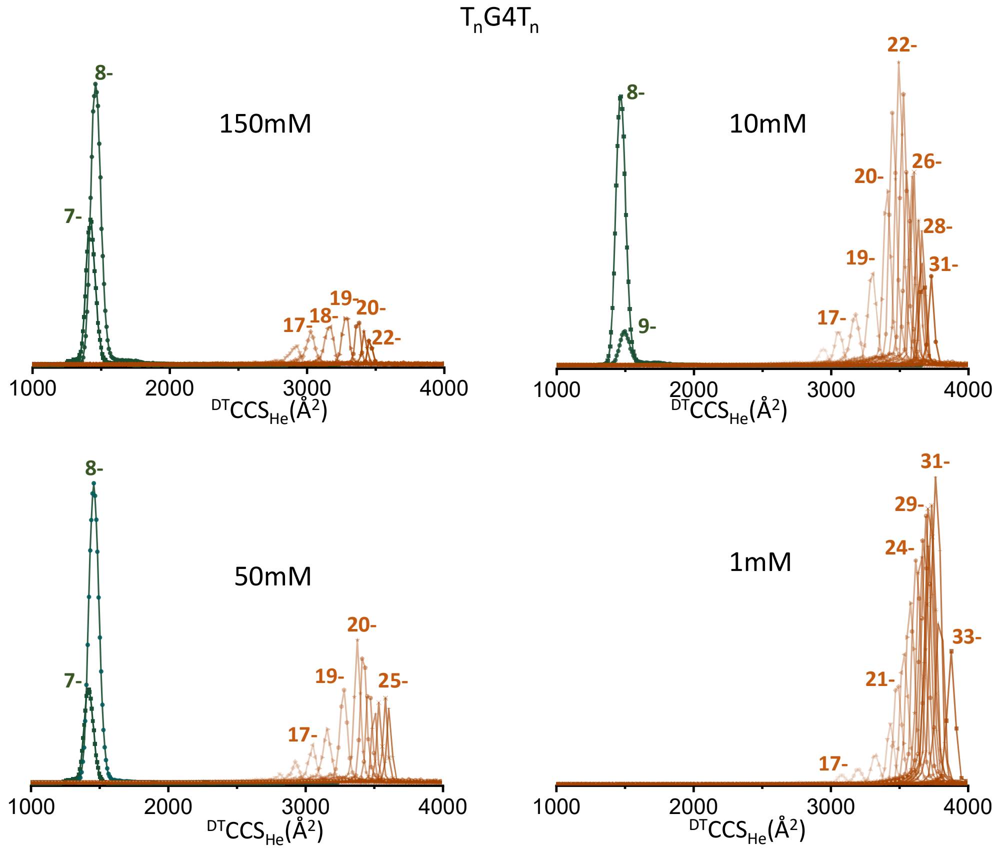

Figure S12: CCSD for ions of TnG4Tn at various ionic strengths showing the absence of third distributions of CCS.

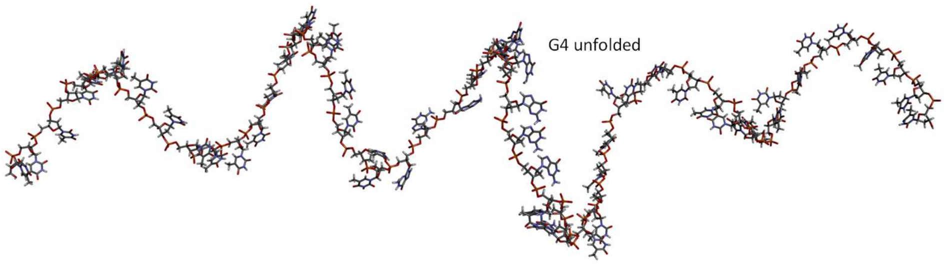

$^{Theo}CCS_{EHSSrot}$ = 3862.7 Å^2

Figure S13: Modelled structure of $(TnG4Tn)^{29-}$ shows the disruption of G4 core before complete elongation of thymine chains.

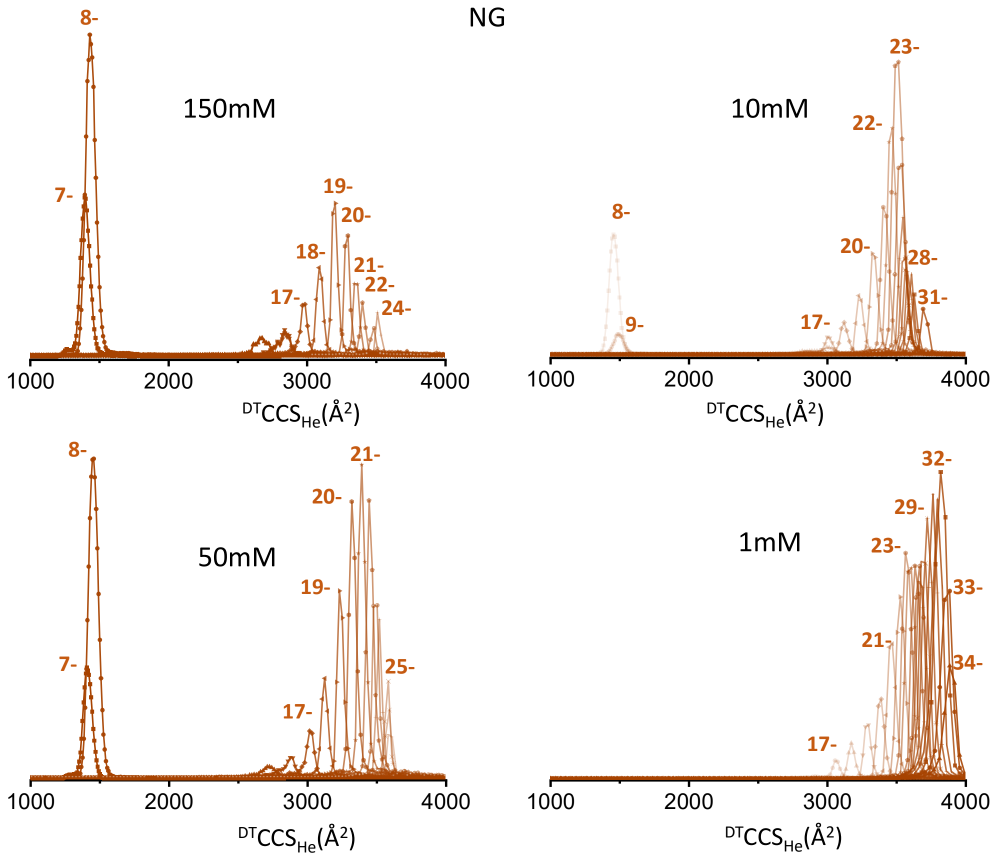

Figure S14: CCSD for ions of NG at various ionic strengths. The relative abundance of extended structures is higher (even at 50 mM ionic strength) in comparison to other 60-mers.

G4T$_n$G4

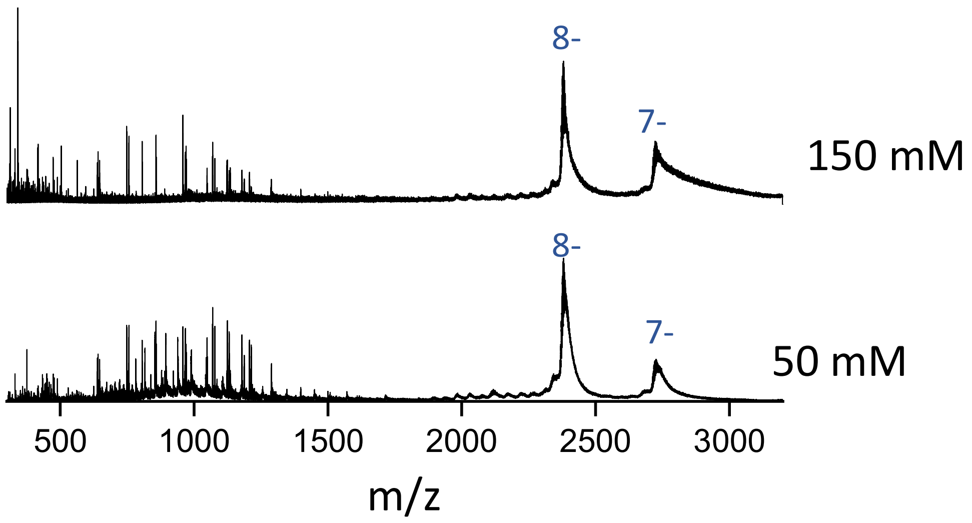

Figure S15: ESI-MS spectra of G4TnG4 at 150 mM and 50 mM aqueous NH_4OAc solution (unfiltered data).

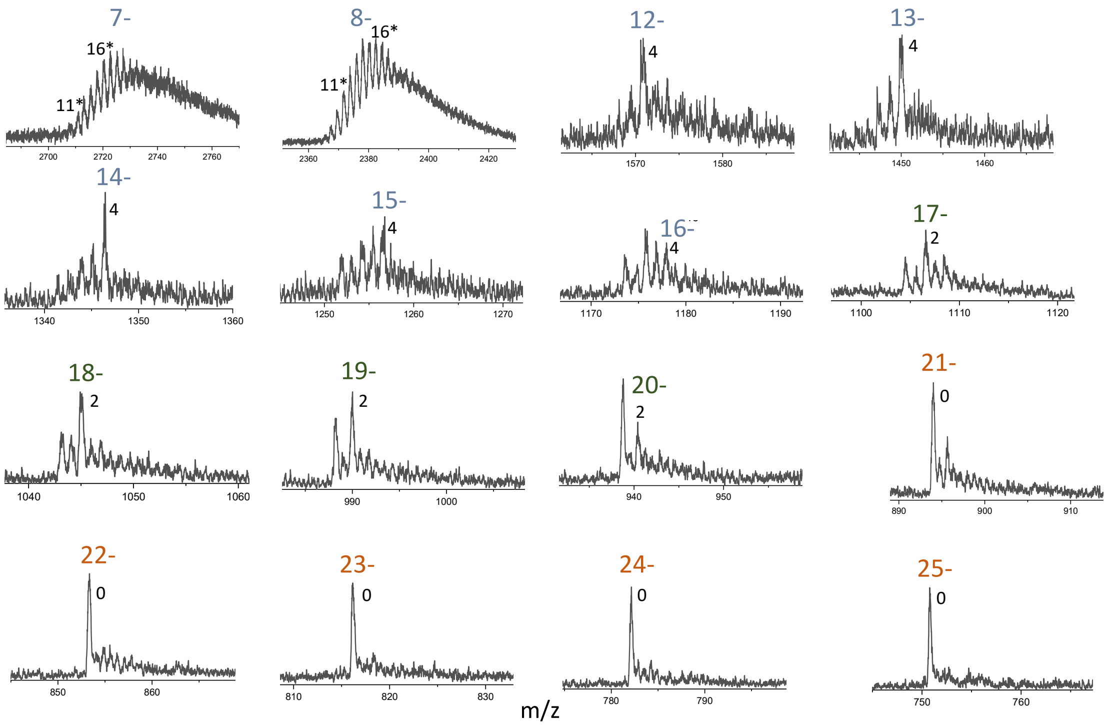

Figure S16: MS of G4TnG4 recorded at 320V show multiple NH_4^+-adducts at low charge states and very few adducts at high charge states in 50 mM NH_4OAc. The numbers indicate the count of non-specific NH_4^+-adducts (marked with an asterisk *), and specific adducts (without asterisk).

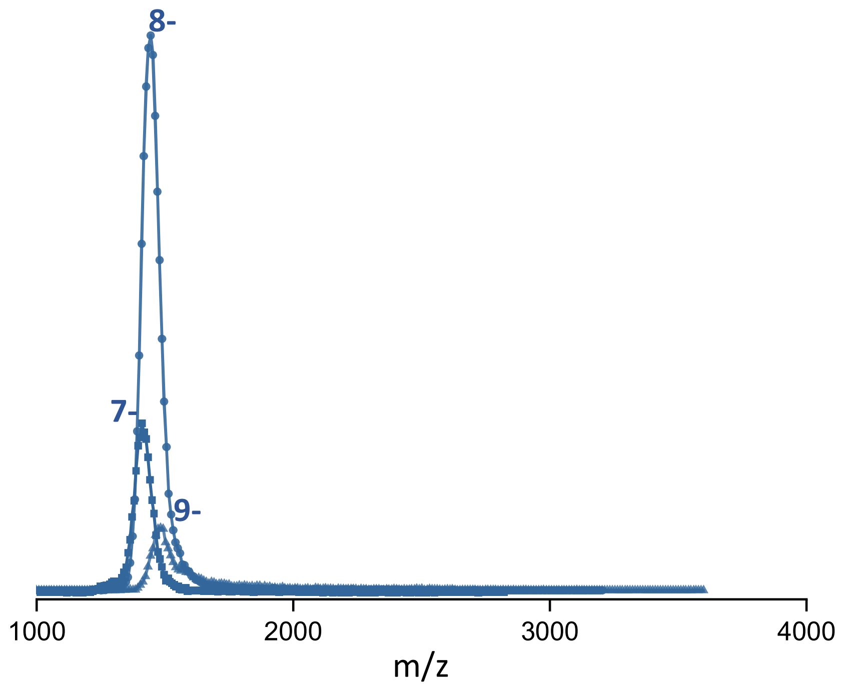

Figure S17: CCSD of G4TnG4 under native supercharging condition in presence of 0.4% of propylene carbonate in 150 mM aqueous NH_4OAc solution showing unimodal distribution with few charge states.

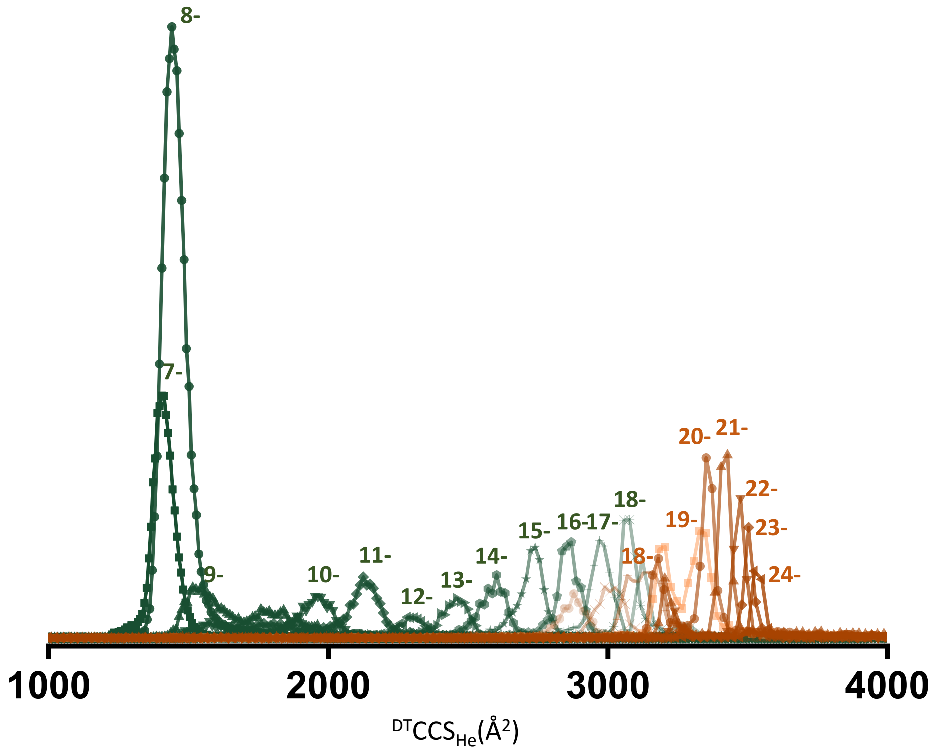

Figure S18: CCSD of G4Tn under native supercharging condition in presence of 0.4% of propylene carbonate in 150 mM aqueous NH_4OAc solution. It exhibits a second distribution of CCS centered on 11- charge state, revealing previously hidden charge states. Under this condition, all four distributions of CCS are clearly observed.

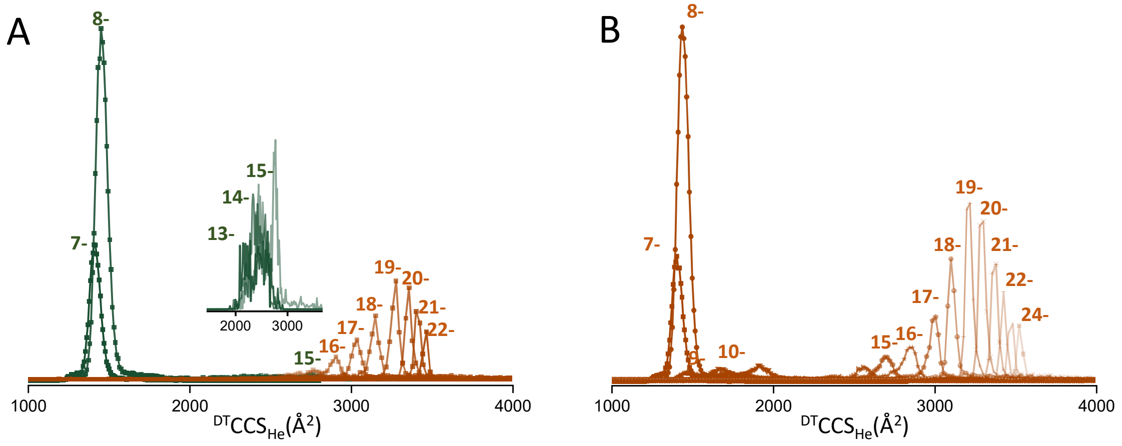

Figure S19: CCSD of TnG4Tn (A), NG (B), under native supercharging condition in presence of 0.4% of propylene carbonate in 150 mM aqueous NH_4OAc solution. Inset in A shows the presence of 13- to 15- ions.

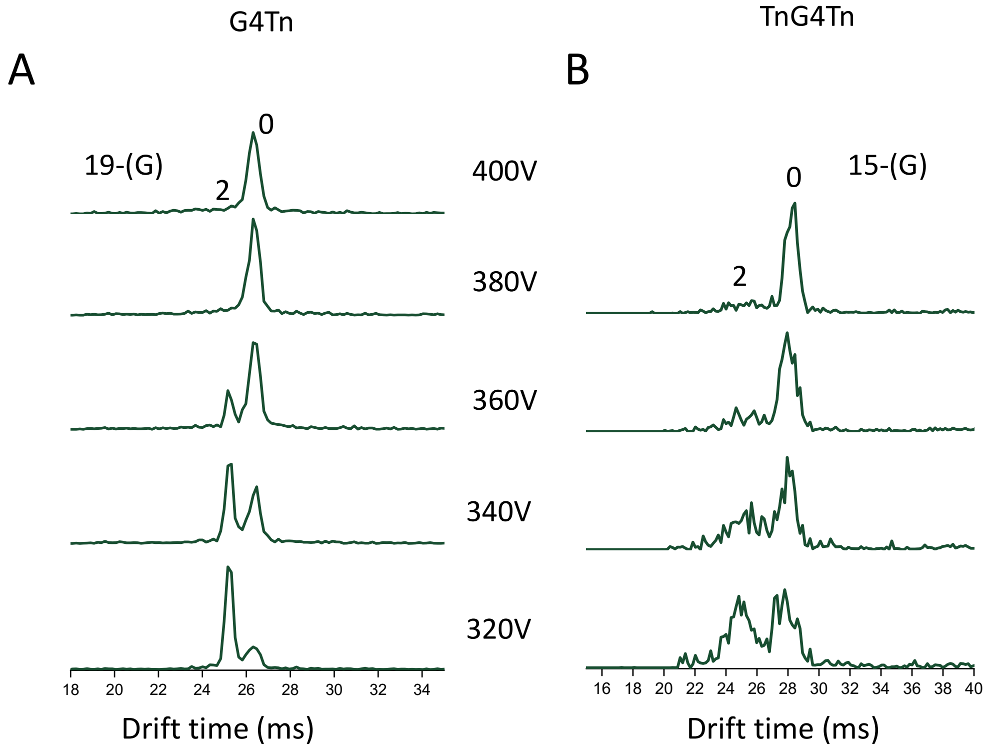

Figure S20: The CIU data: arrival time distributions as a function of the increasing the fragmentor voltage of G4Tn (A) and TnG4Tn (B) in 150 mM aqueous NH_4OAc with 0.4% of propylene carbonate. The numbers indicate the count of specifically bound NH_4^+ ions. At low fragmentor voltage (320V), G4Tn predominantly adopts a folded state at 19- with two specifically bound NH_4^+ ions, whereas TnG4Tn displays two equally abundant distributions at 15-, one with two specifically bound NH_4^+ ions and another lacking NH_4^+ ions. The arrival time distribution plot clearly illustrates the differences in the gas-phase stability of TnG4Tn even for lower charge (15- is shown here) in comparison to G4Tn.

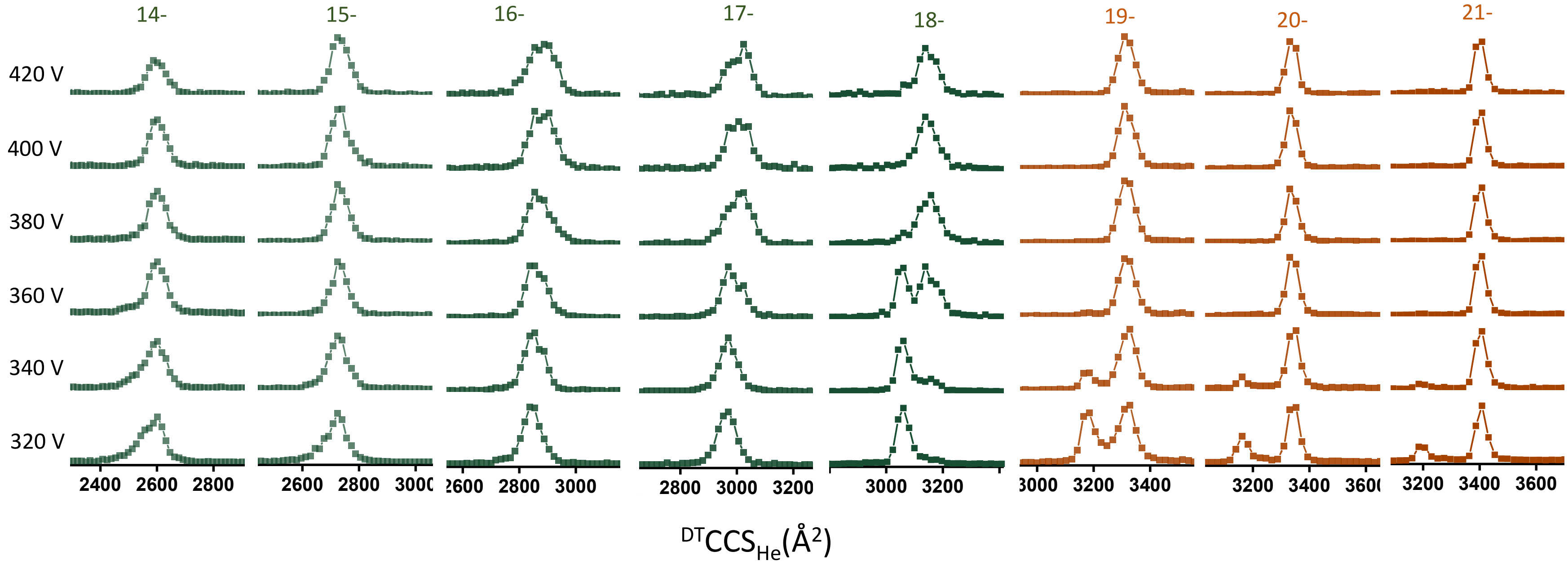

Figure S21: Collision-induced unfolding: evolution of the CCSDs upon increasing the fragmentor voltage (written on the left), for different charge states of G4Tn in 150 mM aqueous NH_4OAc with 0.4% of propylene carbonate.

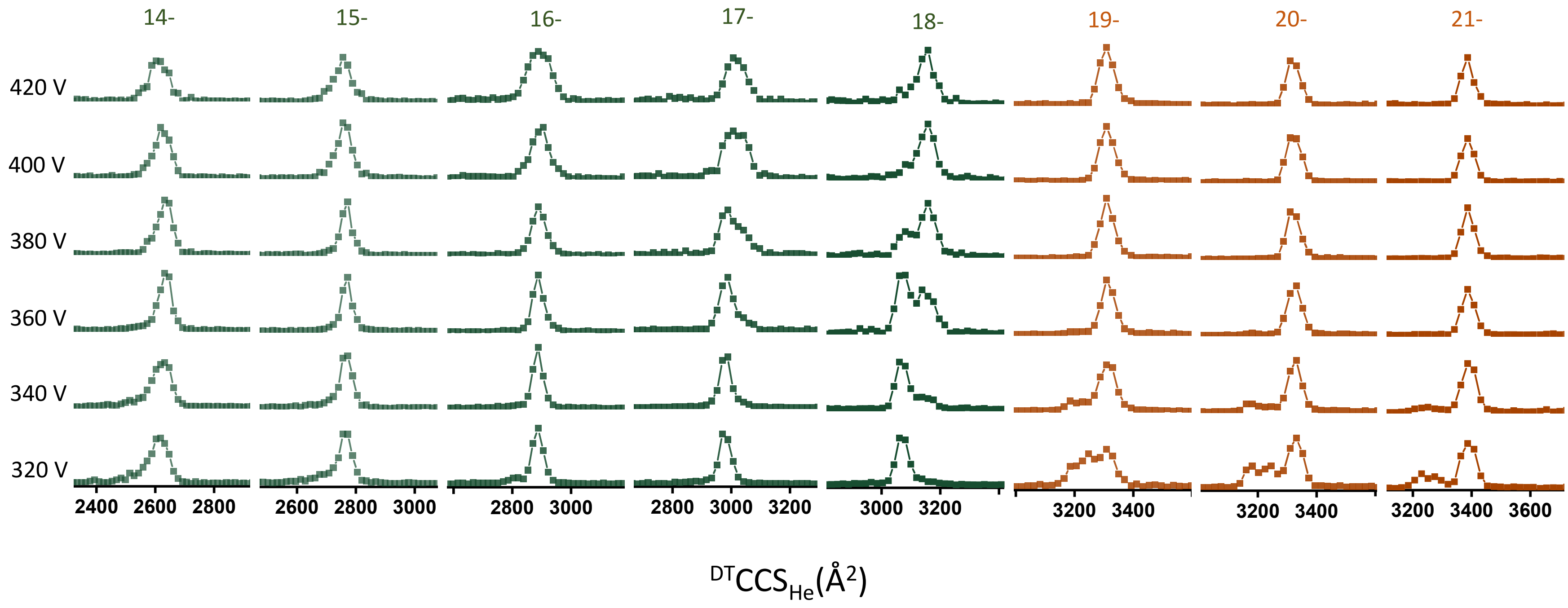

Figure S22: Collision-induced unfolding: evolution of the CCSDs upon increasing the fragmentor voltage (written on the left), for different charge states of TnG4 in 150 mM aqueous NH_4OAc with 0.4% of propylene carbonate.

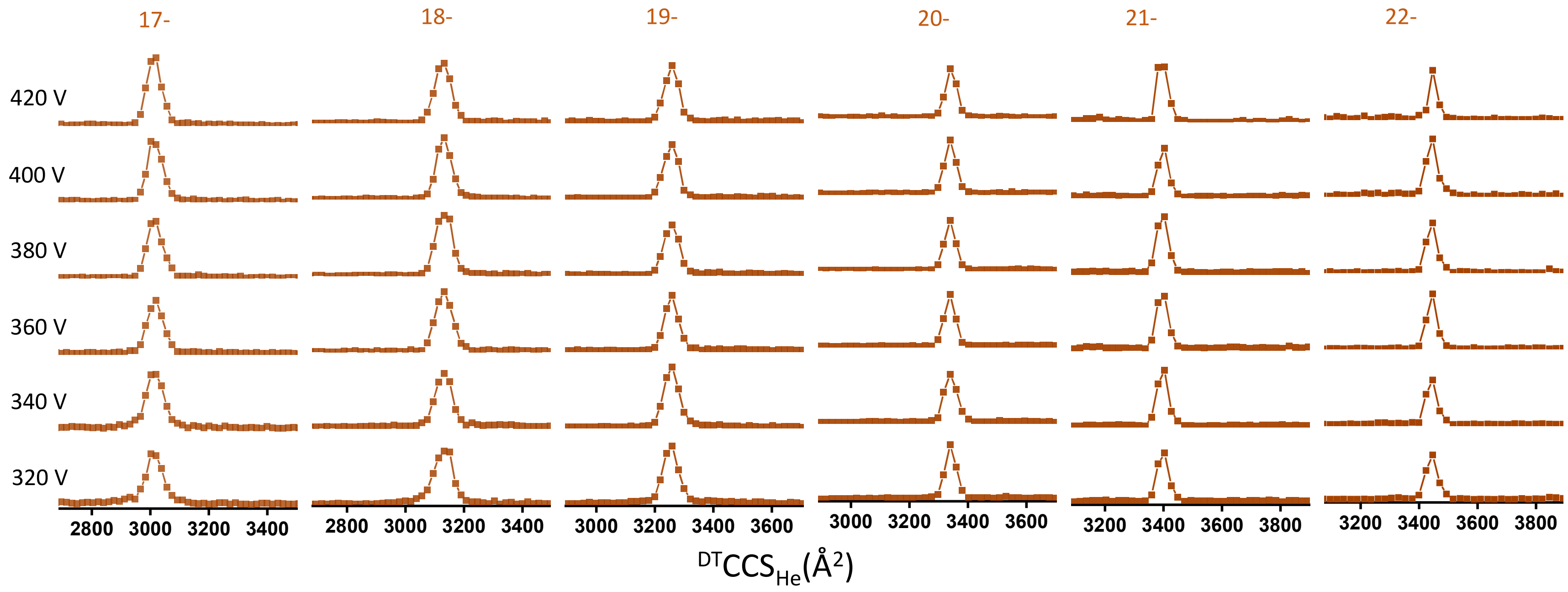

Figure S23: Collision-induced unfolding: evolution of the CCSDs upon increasing the fragmentor voltage (written on the left), for different charge states of TnG4Tn in 150 mM aqueous NH_4OAc with 0.4% of propylene carbonate.

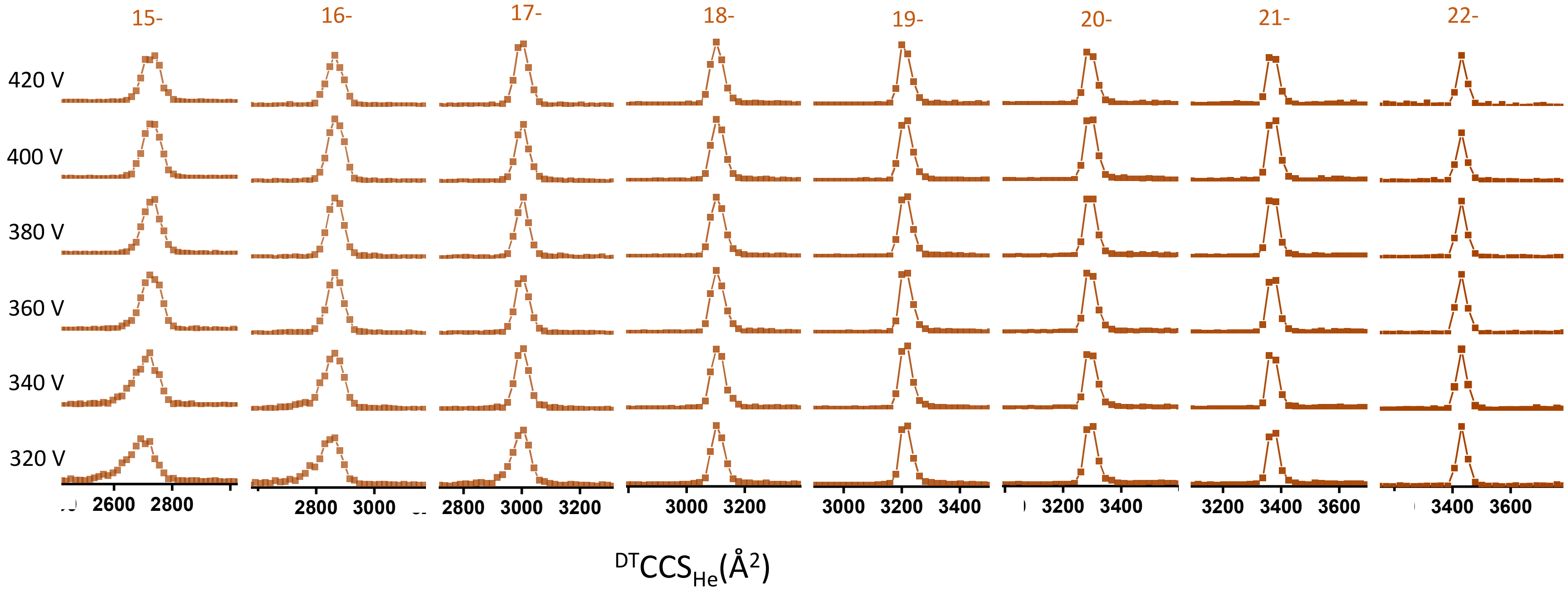

Figure S24: Collision-induced unfolding: evolution of the CCSDs upon increasing the fragmentor voltage (written on the left), for different charge states of NG in 150 mM aqueous NH_4OAc with 0.4% of propylene carbonate.

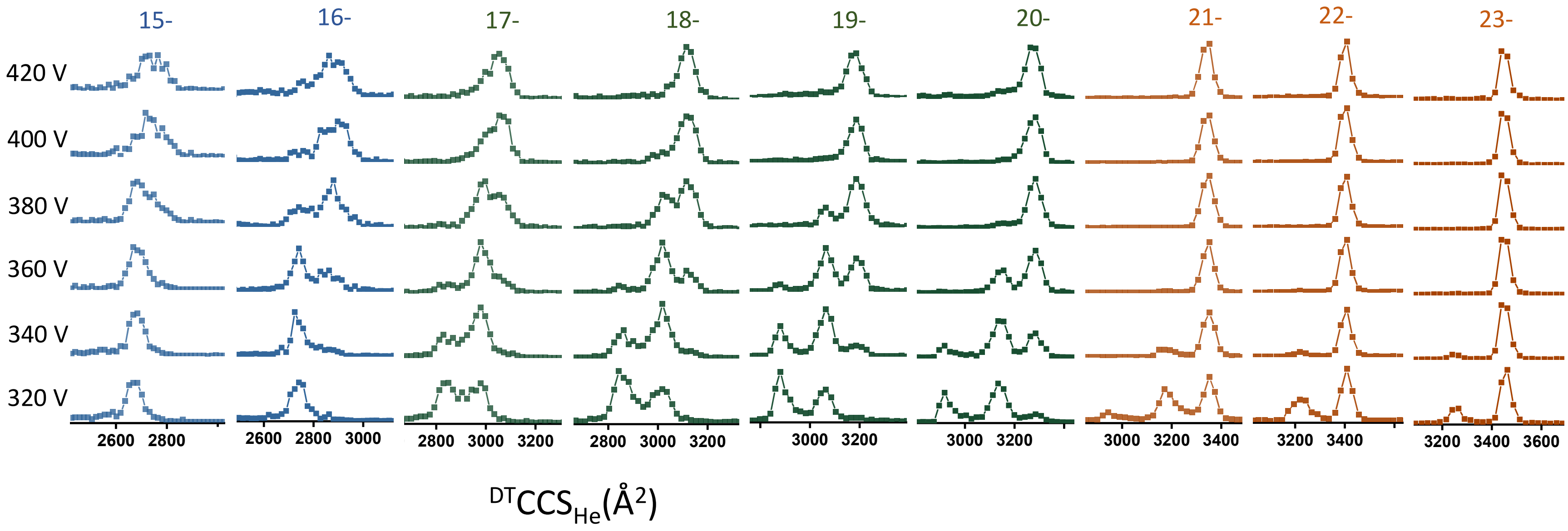

Figure S25: Collision-induced unfolding: evolution of the CCSDs upon increasing the fragmentor voltage (written on the left), for different charge states of G4TnG4 in 50 mM aqueous NH_4OAc with 0.4% of propylene carbonate.

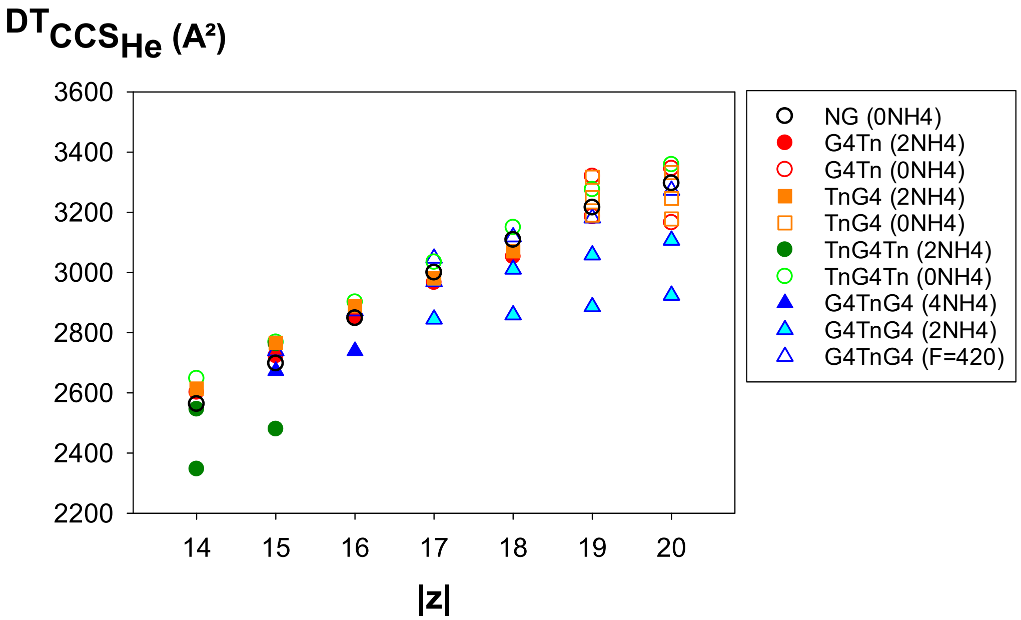

Figure S26: Helium collision cross sections of the 60-mer DNAs, recorded from 150 mM NH_4OAc and 0.4% propylene carbonate, recorded in soft conditions (fragmentor voltage = 320 V), except for G4TnG4 which was recorded in 50 mM NH_4OAc and 0.4% propylene carbonate, and for which the data of the extended forms were taken from data recorded at a fragmentor voltage of 420 V.. Filled symbols correspond to sequences with all ammonium ions and thus with preserved G-quadruplex structures.
